# Editorial: Nutrition for team and individual sport athletes

**DOI:** 10.3389/fspor.2024.1524748

**Published:** 2024-12-02

**Authors:** Alvaro López-Samanes, Gina Trakman, Justin D. Roberts

**Affiliations:** ^1^ GICAF Research Group, Education, Research Methods and Evaluation Department, Faculty of Human and Social Sciences, Universidad Pontificia Comillas, Madrid, Spain; ^2^ Department of Sport, Exercise and Nutrition Sciences, La Trobe University, Melbourne, VIC, Australia; ^3^ Cambridge Centre for Sport and Exercise Sciences, Faculty of Science and Engineering, Anglia Ruskin University, Cambridge, United Kingdom

**Keywords:** dietary supplements, sports nutrition, nutritional periodization, athletes, sports performance

**Editorial on the Research Topic**
Nutrition for team and individual sport athletes

## Introduction

Individual and team sports involve bursts of high-intensity activity interspersed with periods of moderate-lower intensity, but the specific patterns vary across different team sports and individual sports (1). Although it is challenging to predict the exact demands of any given disciplines, nutritional factors play a crucial role in individual or team sport performance, with growing scientific interest in developing personalized nutrition strategies for athletes. Such personalized approaches are designed to support and enhance health, body composition, exercise performance and recovery by tailoring dietary advice to align with an individual's genetic makeup (2) and lifestyle/training demands. However, when implementing personalized sports nutrition programs or incorporating dietary supplements, it is vital to take an evidence-based approach aligned with acknowledged physiological and biochemical mechanisms. Furthermore, evaluating the safety and efficacy of supplements in specific populations and examining their interactions with other components or nutrients are essential considerations. To tackle these topics, we introduced a special issue titled “*Nutrition for Team and Individual Sport Athletes*,” which garnered submissions from researchers and academics experts in the field of sports nutrition. This special issue features fifteen high-quality original research articles. In this editorial, we will provide an overview of the key findings from each manuscript.

## Dietary and nutritional supplements

### Caffeine and beetroot juice

Caffeine and beetroot juice are dietary supplements have been extensively studied in recent years, with good to strong evidence of achieving performance benefits when used in specific scenarios (3). Mor et al., evaluated the impact of moderate-dose caffeine (i.e., 3 mg/kg) on anaerobic performance and hydration status in moderately trained male football players. The results of this study showed that caffeine improved vertical jump capacity and agility without affecting hydration status, but no differences in 30 m sprint, ball kicking speed and balance were found, suggesting caffeine is as an effective ergogenic aid for enhancing some neuromuscular performance determinants in football players. Referring to beetroot juice, Nyman et al., assessed the effectiveness of a mixed beet-based supplement (i.e., beetroot juice) vs. a placebo in reducing inflammation during recovery from sustained intensive cycling (2.25 h) in 20 male and female cyclists, using a multi-omics approach. A randomized, placebo-controlled, double-blind, crossover design was employed. Participants underwent two 2-week supplementation periods with a 2-week washout in between, followed by a 2.25 h cycling exercise at 70% $\dot{V}$O_2max_. The exercise led to an increase in 41 of 67 detected oxygenated polyunsaturated fatty acids (i.e., oxylipins) that are bioactive molecules established as important mediators during inflammation. Beetroot juice supplementation notably raised post-exercise concentrations of anti-inflammatory oxylipins (i.e., 18-HEPE and 4-HDoHE) and proteomic analysis revealed changes in protein clusters related to inflammation. Thus, an acute 2-week intake of a beetroot juice was linked to enhanced post-exercise anti-inflammatory markers and a reduction in protein biomarkers associated with inflammation, supporting its potential in moderating exercise-induced inflammation in athletes.

### Carbohydrate mouth-rinsing

Carbohydrates are the body's main source of energy for athletic events and very used for athletes in several forms (i.e., solid, liquid, mouth rinsing). Nyman et al. study explored the effects of carbohydrate mouth-rinsing on physical performance during ice hockey scrimmages in male athletes. Findings indicated that mouth-rinsing improved endurance and performance metrics during repeated high-intensity efforts on ice hockey performance. Thus, mouth rinsing with carbohydrate solutions (but not consuming) may be a valuable nutritional strategy to protect against decrements in external load with increased playing time in ice hockey without undue negative effects sometimes experienced in carbohydrates ingestions (e.g., gastrointestinal distress).

### Menthol

There is also current interest in menthol consumption, which has been associated with improvements in several aspects of athletic performance including endurance, speed, strength and joint range of motion. Roriz et al. undertook a randomized, counterbalanced crossover design, in which participants consumed either a 0.01% menthol solution or a placebo noncaloric solution 45 min football protocol (i.e., Intermittent Soccer Aerobic Fitness Test). The authors analysed the effects of pre-exercise non-thermal cooling sensations on examined perceptual, physical, and physiological responses in football referees in a protocol consisting of two 45 min intermittent exercise bouts under hot and humid conditions, with physical performance measured as the total distance covered across three 15 min exercise blocks. Mouth rinsing with a menthol solution resulted in immediate improvements in thermal sensation and comfort. However, these perceptual benefits did not persist throughout the 45 min exercise protocol and did not translate into improvements in physical performance or physiological responses. Therefore, based on the findings, use of menthol solutions may be of limited impact for sustained events.

### Turmeric

Acute muscle damage and subsequent inflammatory responses associated with intensive exercise has led to interest in strategies to promote rapid recovery which may be paramount to optimising subsequent performance and reducing injury risk. For this reason, Clayton et al., undertook a between-groups design study design to examine the effect of a turmeric supplement (i.e., 60 ml turmeric drink twice per day), containing high concentrations of curcumin a polyphenol proposed to reduce muscle damage and soreness in recreational athletes. The study aimed to determine whether turmeric supplementation could enhance recovery and improve neuromuscular performance [e.g., countermovement jump (CMJ) and isometric mid-thigh pull (IMTP)] and physiological markers of recovery [e.g., creatine kinase (CK) and plasma C-reactive protein (CRP)] in elite male football players. Measurements were taken immediately (0 h) and at 40–64 hours post-match following eight competitive matches showing that the curcumin-containing supplementation appeared to attenuate post-match inflammation, as indicated by reduced CRP levels. However, no significant effects were found on muscle damage markers (CK) or on neuromuscular performance measures (CMJ and IMTP). As such, further research is needed to establish the acute and chronic effects of polyphenol nutrients such as curcumin.

### Other nutrients

New dietary or nutritional supplements appear in the scientific domain constantly and there is need to verify their efficacy. According to Penggalih et al., marine-derived substances, such as omega-3 fatty acids, proteins, biopeptides, carotenoids, glucosamine, and minerals, have demonstrated potential in addressing obesity-related health issues, including dyslipidaemia, diabetes, oxidative stress, and inflammation. However, their effects on sports performance are not as well-documented. For this reason, the authors of this review stress the importance of further research to investigate marine-derived proteins and the development of functional foods specifically designed for high-performance athletes. In addition, Zhou et al., undertook a systematic review and meta-analysis regarding effects of molecular hydrogen (H₂) supplementation and potential contribution in enhancing physical performance (i.e., endurance, muscular strength, and explosive power performance). Authors stated that H₂ supplementation short-term (<14 days) enhance lower limb explosive power, relieve fatigue, and improve blood lactate clearance, although they may not significantly boost aerobic endurance, anaerobic endurance, or muscular strength, being inhalation of H₂ appears to be the most effective method for enhancing physical performance, particularly lower limb explosive power, in healthy adults. Future studies with robust designs are needed to provide more definitive conclusions regarding the effects of H₂ on lower limb explosive power and muscle strength in this population.

### Perception and use of supplements

The abovementioned studies support the utility of some supplements, when used in defined athletic populations (including football players), to support discrete performance outcomes. Acknowledging that research on supplements is a growing field, Abreu et al., aimed to investigate the supplement perspectives and practices of nutritionists working with elite football teams, via online questionnaire. They established that the majority of nutritionist (70.8%) agreed or strongly agreed to recommend dietary supplements to football players. Interesting, however, just 50% of nutritionist believed that supplements were effective, which may reflect findings in the literature, including within this special issue, that efficacy varies across supplements, sporting discipline, and outcome of interest.

### Relationships between body composition, nutritional status/intake, health status (including RED-s), and nutritional knowledge assessment

Oukheda et al. evaluated whether the dietary practices of professional and adolescent football players in Morocco during the competitive period met international macronutrient recommendations and explored the relationship between their nutritional status and aerobic performance, as measured by the Yo-Yo IRL1 test. The authors reported that higher intake levels of carbohydrates and proteins were positively correlated with the total distance covered by the players, while higher proportion of energy derived from fats in the diet was negatively correlated with the distance covered. Therefore, optimizing carbohydrates and protein intake while managing fat consumption may be highly important for enhancing football performance. Staśkiewicz et al. assessed changes in body composition among professional football players throughout the macrocycle season, aiming to identify the correlation between nutrition knowledge and the maintenance of muscle mass. The authors assert that players’ knowledge of macronutrient subcategories was significantly negatively correlated with the variability of skeletal muscle mass content. This finding establishes that nutrition knowledge impacts the stability of body composition across all analysed periods: preparatory, competitive, and transition phases highlighting the benefits of targeted strategies to improve the level of nutritional knowledge of athletes to maintain appropriate body composition. Ostensibly, the relationship between knowledge and body composition identified by Staśkiewicz et al. is mediated by dietary practices. AlKasasbeh and Akroush, in a cross-sectional study, aimed to explore the relationships between food habits, perceived barriers to healthy eating, and sports nutrition knowledge among adolescent swimmers. The study found a significant positive association between food habits and sports nutrition knowledge, determining that nutrition knowledge emerged as a significant positive predictor of healthy food habits. Nicholas and Grafenauer, were also interested in the potential value of knowledge and education. Their research aimed to establish a baseline understanding of dance students at a single pre-professional institution, using metrics focused on current health, nutrition, lifestyle, and overall wellbeing, while also assessing their knowledge of long-term health implications. Utilizing a cross-sectional study design, the Dance-Specific Energy Availability Questionnaire was adapted for Australian participants and administered online. They determined that assessing the health status and preventative health knowledge of pre-professional dancers can inform educational strategies that promote dancers’ health and career longevity. The findings provide valuable insights into health knowledge and specific issues relevant to dancers, underscoring the need for tailored educational strategies to emphasize preventative health. Additionally, the study highlights the necessity for increased awareness of low energy availability and relative energy deficiency (REDs) in sport among practitioners working with dancers, as well as cultural and structural changes within the wider dance community to safeguard and enhance dancers’ wellbeing. Also in the REDs field, Dvořáková et al. provide a comprehensive review of the markers used to diagnose Relative Energy Deficiency in Sport REDs and compare them to the REDs CAT2 score. This review highlights the most used diagnostic markers, such as bone mineral density, anthropometric parameters, and T3 hormone concentration. The authors emphasize the importance of standardizing methodologies in future research to assess the holistic nature of an individual's profile in determining the presence of REDs or other contributing factors as previously suggest in the literature (4).

### Wider aspect of diet and nutrition

Finally, the last two articles of our special issue covered wider fields such as: the impact of nutrition on visual perceptual-cognitive performance in healthy adults; the interrelationships among food habits, sports nutrition knowledge, and perceived barriers to healthy eating in adolescent swimmers; and association between diet and sleep with internalising symptoms in young athletes. Beathard et al., examined the impact of nutritional intake on visual perceptual-cognitive performance in young, healthy adults. The authors reported that cognitive function benefits from higher dietary intake of carbohydrates, lutein/zeaxanthin, and vitamin B2. Conversely, elevated protein consumption was associated with a negative effect on visual perceptual-cognitive performance in female participants. Finally, Gao and Wang, realized a cross-sectional study investigating the association between diet, sleep, and internalizing symptoms in 758 young Chinese athletes. The Australian Athletes Diet Index was employed to evaluate dietary patterns, while sleep quality was measured using the Athletes Sleep Screening Questionnaire. Symptoms of anxiety and depression were assessed using the Generalized Anxiety Disorder 7 scale and the 9-item Patient Health Questionnaire. The findings indicated that diet mediated the relationship between chronotype and sleep quality, while sleep quality mediated the association between diet and symptoms of anxiety and depression. Furthermore, both diet and sleep quality jointly mediated the association between chronotype and symptoms of anxiety and depression.

## References

[1] Mujika I Burke LM. Nutrition in team sports. Ann Nutr Metab. (2011) 57(Suppl 2):26–35. 10.1159/000322700 21346334

[2] Guest NS Horne J Vanderhout SM El-Sohemy A. Sport nutrigenomics: personalized nutrition for athletic performance. Front Nutr. (2019) 6:8. 10.3389/fnut.2019.00008 30838211 PMC6389634

[3] Maughan RJ Burke LM Dvorak J Larson-Meyer DE Peeling P Phillips SM Ioc consensus statement: dietary supplements and the high-performance athlete. Br J Sports Med. (2018) 52(7):439–55. 10.1136/bjsports-2018-099027 29540367 PMC5867441

[4] Jeukendrup AE Areta JL Van Genechten L Langan-Evans C Pedlar CR Rodas G Does relative energy deficiency in sport (REDs) syndrome exist? Sports Med. (2024) 54:2793–816. 10.1007/s40279-024-02108-y 39287777 PMC11561064

